# Staged use of ordinal and linear disability scales: a practical approach to granular assessment of acute stroke outcome

**DOI:** 10.3389/fneur.2023.1174686

**Published:** 2023-06-28

**Authors:** Napasri Chaisinanunkul, Sidney Starkman, Jeffrey Gornbein, Scott Hamilton, Fiona Chatfield, Robin Conwit, Jeffrey L. Saver

**Affiliations:** ^1^Stroke Center, Phyathai 1 Hospital, Bangkok, Thailand; ^2^Comprehensive Stroke Center and Departments of Emergency Medicine and Neurology, University of California, Los Angeles, Los Angeles, CA, United States; ^3^Department of Biomathematics, University of California, Los Angeles, Los Angeles, CA, United States; ^4^Department of Neurology, Stanford University, Palo Alto, CA, United States; ^5^Comprehensive Stroke Center and Department of Neurology, University of California, Los Angeles, Los Angeles, CA, United States; ^6^National Institutes of Health, Bethesda, MD, United States; ^7^Indiana University School of Medicine Department of Neurology, Indianapolis, IN, United States

**Keywords:** cerebrovascular disease/stroke, acute cerebral hemorrhage, acute cerebral infarction, acute stroke syndromes, emergency treatment of stroke, outcome assessment, clinical trials

## Abstract

**Background:**

The modified Rankin Scale (mRS) assessment of global disability is the most common primary endpoint in acute stroke trials but lacks granularity (7 broad levels) and is ordinal (scale levels unknown distances apart), which constrains study power. Disability scales that are linear and continuous may better discriminate outcomes, but computerized administration in stroke patients is challenging. We, therefore, undertook to develop a staged use of an ordinal followed by a linear scale practical to use in multicenter trials.

**Methods:**

Consecutive patients undergoing 3-month final visits in the NIH FAST-MAG phase 3 trial were assessed with the mRS followed by 15 mRS level-specific yes–no items of the Academic Medical Center Linear Disability Score (ALDS), a linear disability scale derived using item response theory.

**Results:**

Among 55 patients, aged 71.2 (SD ± 14.2), 67% were men and the entry NIHSS was 10.7 (SD ± 9.5). At 90 days, the median mRS score was 3 (IQR, 1–4), and the median ALDS score was 78.8 (IQR, 3.3–100). ALDS scores correlated strongly with 90 days outcome measures, including the Barthel Index (*r* = 0.92), NIHSS (*r* = 0.87), and mRS (*r* = 0.94). ALDS scores also correlated modestly with entry NIHSS (*r* = 0.38). At 90 days, the ALDS showed greater scale granularity than the mRS, with fewer patients with identical values, 1.9 (SD ± 3.2) vs. 8.0 (SD ± 3.6), *p* < 0.001. When treatment effect magnitudes were small to moderate, projected trial sample size requirements were 2–12-fold lower when the ALDS rather than the mRS was used as the primary trial endpoint.

**Conclusion:**

Among patients enrolled in an acute neuroprotective stroke trial, the ALDS showed strong convergent validity and superior discrimination characteristics compared with the modified Rankin Scale and increased projected trial power to detect clinically meaningful treatment benefits.

## Introduction

The modified Rankin Scale (mRS) assessment of global disability is the most widely employed primary endpoint in acute stroke clinical trials (1–5). However, the mRS has two structural constraints that potentially limit its power to detect clinically meaningful treatment effects: coarseness and ordinality.

The mRS arrays patients among only seven broad scales, from normal, through five degrees of disability, to dead (6). This lack of granularity makes the scale less responsive - unable to detect moderate changes that are relevant to patients, families, and society (7–9). For example, although their level of functioning is very different, the scale may assign the same score of 2 to a patient with work-limiting post-stroke fatigue but with no focal neurologic deficits and fully normal gait and to a patient who can ambulate only slowly with a walker. In addition, the mRS is an ordinal scale without an internal definition of distances from one level to another, whether going from a 5 to a 4 is the same, better, much better, worse, or much worse than going from a 2 to a 1 is not defined by scale construction. The ordinal character of the mRS limits its statistical analytic power, a limitation only partially mitigated by assigning utility weights to the levels of the mRS (10).

Disability scales that are more fine-grained and that are interval, rather than ordinal, may be more informative than the mRS. The Academic Medical Center Linear Disability Score (ALDS) is a candidate instrument with these properties. The ALDS scale is a calibrated, generic item bank to measure the level of physical disability in patients (11–13). The ALDS draws upon 77 items to array patients on a single, hierarchical linear scale, developed using the item response theory. The ALDS was designed as a generic measure applicable to all disease states, has been deployed in a proof of principle non-trial stroke population, and has been recognized by a consensus group as a promising potential instrument for use in stroke trials (14). However, conventional administration of the ALDS uses an adaptive, computerized methodology that is challenging to employ in stroke patients and in multicenter clinical trials (15–17). A hybrid testing approach is a two-stage process with an initial broad classification tool that guides short-form selection for more detailed grading (18). This study was undertaken to compare the mRS and the ALDS in a population of stroke patients actually enrolled in an acute stroke trial and to assess how the observed scale performance characteristics would affect projected clinical trial power.

## Methods

### Subjects

The study population consisted of 55 consecutive patients enrolled in the NIH Field Administration of Stroke Therapy—Magnesium Phase 3 Trial (registration number NCT0005933) assessed at 90 days by study coordinators certified in the mRS and trained in the ALDS. The FAST-MAG enrollment criteria included that subjects be aged 40–95 years, have signs of likely acute stroke when assessed in the ambulance, and be within 2 h of last known well time (8).

### Assessments

At enrollment, pretreatment stroke severity was assessed by paramedics using the Los Angeles Motor Scale (LAMS) (9), and early post-ED arrival stroke severity was assessed by certified study investigators or coordinators using the NIH Stroke Scale (NIHSS).

At 90 days, subjects were assessed with the NIH Stroke Scale (neurologic deficit), the Barthel Index (activities of daily living), the modified Rankin Scale, and the AMC Linear Disability Score. Among the two disability instruments, the mRS was performed first, before the ALDS. For the mRS, the Rankin Focused Assessment was employed to operationally assign modified Rankin Scale grades (10). Whenever possible, information on patient functioning was obtained both from the patient and from available relatives and caregivers. If patients were unable to communicate effectively due to language or other cognitive disorders, the relative or healthcare proxy was the main information source. Assessments were performed in person whenever possible, with phone ascertainment of the mRS and the ALDS when an in-person encounter could not be arranged.

### ALDS item sets

Unlike sum-score-based methods, item banks administer only a subset of items to each subject, tailored to each patient's level of functioning. By selecting items enriched at the region in the scale in which the patient's disability level falls, item banks can efficiently quantify patient function. The overall ALDS item bank consists of 77 items quantifying disability status, ranging from relatively easy to difficult.

For this study, from the overall bank, we constructed 5 ALDS question sets, each containing 15 items, indexed to within, above, and below the mRS score (see Supplementary Methods Text). For example, if the patient had an mRS of 2, then the rater administered the ALDS 2 question set; if the patient had an mRS of 3, then the rater administered the ALDS 3 question set. Across all five test sets, a total of 35 ALDS items are used. The 35 items span the entire range of ALDS quantified disability. The item suites on ALDS sets 1–5 were generally selected to cluster at particular points along the disability spectrum, centered on the average performance for each mRS level observed in the proof of principle study (7), but also to include a few lower and higher items to capture outlying cases in which the ALDS score might place the patient at a level quite discrepant with the mRS score. As a result, each ALDS question set includes not only multiple ALDS questions that typically fall within the range of the patient's particular mRS score but also outlier questions that typically fall at levels 1 to 2 mRS steps higher and 1 to 2 mRS steps lower than the patient's particular mRS score. This approach permits ALDS scoring to be potentially discrepant with mRS scoring, as might occur if an inexperienced rater assigns a patient a non-consensus mRS score. Additionally, raters were instructed that, if every question in the initial ALDS question set ends up marked all “yes” or all “no”, they should proceed to also administer the next lower or higher numbered ALDS question set.

Raters received a single 30-min training session in administering the ALDS. Due to its simple yes–no question structure, more detailed training was deemed unnecessary.

### Statistical analysis

ALDS logit scores for each patient were derived from their responses to administered test items using previously published item measures and maximum likelihood methods, following previously described methods (8). In addition, resulting scores were transformed to a 0–100 scale, computed using the RASCH logit model: Transformed score = [exp(logit score)/1 + exp (logit score)] x 100.

ALDS scores were assessed for floor and ceiling effects by analyzing the percentage of patients with maximal or minimal scores. Internal validity of the ALDS in the stroke population was assessed by examining whether the probability of performance of items among stroke patients correlated with the probability of performance of items in the scale's derivation population. Concurrent validity was assessed by measuring the extent to which the ALDS correlated with the mRS, a direct measure of disability. Convergent validity was assessed by measuring the extent to which the ALDS correlated with the BI and NIHSS, which assess related aspects of stroke recovery. Scale granularity vs. the mRS was assessed by analyzing the number of subjects with identical scale scores.

Correlations among scales were assessed using Spearman's correlation coefficients (*r*_*s*_). Strength of association was categorized as follows: correlation coefficients *r*_*s*_ = 0.00–0.19 as very weak, *r*_*s*_ = 0.20–0.39 as weak, *r*_*s*_ = 0.40–0.59 as moderate, *r*_*s*_ = 0.60–0.79 as strong, and *r*_*s*_ = 0.80–1.00 as very strong. Statistical significance was determined using the two-sided tests.

### Sample size of patients administered the ALDS

This study was a supplementary investigation for the main FAST-MAG trial, undertaken in a subset of patients with a sample size constrained by the need to minimize patient and coordinator burden to ensure parent trial protocol adherence. Formal sample size calculations for determining a statistically significant correlation coefficient between the ALDS and the mRS at day 90, using two-sided testing with 90% power and minimal expected correlation of 0.80 were 20 (16). The size was increased to 55 based on the investigator's judgment of the size that the best-balanced exploration of the increased precision of the ALDS vs. the mRS with minimizing parent trial burden.

### Projected trial sample sizes

Based on the observations in actual patients, sample sizes were computed for trials in which interventions exerted different magnitudes of treatment effect corresponding to mRS shifts of 0.1, 0.2, 0.3, 0.4 0.5, 0.6, 0.7, 0.8, 0.9, and 1.0. Sample size projections used the nonparametric Wilcoxon rank sum test, with a power of 0.80 and a two-sided alpha of 0.05. Splines and linear regression were used to determine the nature of the relationship between mRS and the ALDS logit score using the original data. To derive an ALDS logit value for fatal outcomes (mRS 6), the average decrease in the ALDS logit for each one-unit increase in mRS value over the 0–5 range (2.8 logit units per mRS unit) was added to the logit value for mRS 5. The ALDS logit value corresponding to an mRS of 6 was computed by extrapolating one mRS unit.

The actual mRS and logit ALDS values in the original dataset were used as the “control” group. From this dataset, mRS data for an “experimental” group was artificially generated for a given true mRS “shift” (mean difference) of size Δ, with values of Δ set to 0.1, 0.2, 0.3, 0.4 0.5, 0.6, 0.7, 0.8, 0.9, and 1.0. Within a given resampling iteration, for the ALDS logit scores, the value of 2.8 times Δ was added to the control values to generate shifted values for the experimental group.

For the mRS scores, the score for individual experimental group patients was generated by first generating a uniform random value “U” between 0 and 1 for the sampled control group patients. If U was less than the threshold of Δ, then the experimental group patient score was generated as 1 mRS point lower than the control patient score. Otherwise, the experimental group patient score was the same as the control patient score. The mean mRS difference between the two groups is therefore Δ. Δ is the mean amount of “improvement” in mRS in the experimental group compared to the control group.

Once the experimental group was created, the sample size was computed based on at least 5,000 “bootstrap” resamplings. In each bootstrap iteration, we sampled independently from the control and experimental group to create two unpaired samples and compared them *via* the non-parametric Wilcoxon rank sum test. The sample size needed for 80% power was computed separately for the RS shift and the corresponding shift in the ALDS logit score.

This comparative study of the ALDS and the mRS was approved by the Institutional Review Board of the University of California, Los Angeles.

## Results

The baseline characteristics of the 55 patients are shown in Table 1. Notably, the mean age was 71.2 (SD ± 14.2), 67% were men, and early post-ED arrival NIHSS was a mean of 10.7 (SD ± 9.5). The 55 patients were enrolled in 48 different ambulances and received acute care at 25 different hospitals. Cerebrovascular disease subtype diagnosis was acute ischemic stroke in 65.5%, transient ischemic attack in 20%, and intracranial hemorrhage in 14.5%. At 90 days, 48 patients were alive and 7 were dead. The median mRS was 3 (IQR, 1–4), the median NIHSS score was 2.5 (IQR, 0–8.5), and the median BI score was 95.0 (IQR, 36–100).

**Table 1:** Baseline characteristics.

**Characteristics**	**Mean (SD) OR *N* (%)**
Age, y	71.2 (14.2)
Male sex	37 (67.3%)
**Stroke subtype**	
TIA	11 (20%)
Ischemic stroke	36 (65.5%)
Intracerebral hemorrhage	8 (14.5%)
**Race**	
White	45 (81.8%)
Black	8 (14.5%)
Asian	1 (1.8%)
American Indian/Alaskan Native	1 (1.8%)
**Ethnicity**	
Non-Hispanic	50 (90.9%)
Hispanic	5 (9.1%)
Los Angeles Motor Scale	3.67 (1.20)
**LAMS Severity Categories**	
0–3	24 (43.6%)
4–5	31 (56.4%)
Early NIHSS	10.7 (9.5%)
**NIHSS Severity Categories**	
0–5 (minor)	20 (36.4%)
6–13 (moderate)	21 (38.2%)
≥14 (major)	14 (25.5%)

At day 90, among the 48 survivors, the median ALDS logit score was 2.0 (IQR 0–9.55, range (−13.2 to 9.6) and the median transformed ALDS score was 87.9 (IQR 49.9–100, range 0–100). After assigning the ALDS logit score of −7.7 to the patients with fatal outcomes, across all 55 patients, the median ALDS logit score was 1.3 (IQR −3.4 to 9.6, range −13.2 to 9.6) and the median transformed ALDS score was 78.8 (IQR 3.3–100, range 0–100). Table 2 shows the probability that patients in each mRS grade were able to perform increasingly difficult ALDS items. The ALDS was free of floor effects. Only one patient with an mRS of 5 could not perform any of the 15 least difficult items and had the lowest possible ALDS score. A ceiling effect was noted, with 18 patients (11 with mRS 0 and 7 with mRS 1) able to perform all of the 15 most difficult items and attain the highest possible ALDS score. The internal validity of the ALDS was excellent, with the correlation between the percentage of study patients performing a scale item and that item's previously assigned disability value being *r* = 0.93.

**Table 2:** Probability that patients in each mRS grade were able to perform increasingly difficult ALDS items.

**Item content**	**MRS0 (%)**	**MRS1 (%)**	**MRS2 (%)**	**MRS3 (%)**	**MRS4 (%)**	**MRS5 (%)**
1. Can you vacuum a flight of stairs?	91.7	83.3	33.3			
2. Can you carry a bag of shopping upstairs?	100	91.7	66.7			
3. Can you go for a walk in the woods?	100	91.7	100			
4. Can you travel by local bus?	91.7	91.7	100	11.1		
5. Can you carry a tray?	100	91.7	33.3	44.4		
6. Can you walk up a hill?	100	91.7			0	
7. Can you go shopping for clothes?			100			
8. Can you cut your toe nails?	100	91.7	33.3	66.7		
9. Can you stand for 10 min?	100	100	100	88.9		
10. Can you use a washing machine?	91.7	100	100			
11. Can you walk up a flight of stairs?					0	
12. Can you walk down a flight of stairs?	100	91.7	100	66.7		
13. Can you go for a short walk (15 min)?	100	100		88.9		
14. Can you change the sheets on a bed?	100	100	66.7	33.3		0
15. Can you buy a few things from the store?	100	100	100		16.7	
16. Can you take a shower and wash your hair?	100	100	66.7	88.9	16.7	
17. Can you pick something up from the floor?	100	100	66.7	66.7	33.3	16.7
18. Can you get in and out of a car?				100	16.7	0
19. Can you peel and core an apple?					0	0
20. Can you prepare breakfast or lunch?			100	66.7	16.7	
21. Can you eat a meal at the table?					100	83.3
22. Can you put on/take off socks and slippers?				66.7	33.3	0
23. Can you sit up (from lying) in bed?						16.7
24. Can you get a book off the shelf?						16.7
25. Can you answer the telephone?						50
26. Can you make a bowl of cereal?						0
27. Can you put pants on?					16.7	
28. Can you sit on the edge of a bed from lying down?				100	50	
29. Can you move between two dining chairs?						0
30. Can you wash and dry your lower body?						16.7
31. Can you put on and take off a coat?				88.9	16.7	0
32. Can you wash and dry your face and hands?						50
33. Can you get out of bed into a chair?						0
34. Can you walk to and get on and off the toilet?				88.9	16.7	
35. Can you wash your lower body when taken to sink?					50	

The convergent validity of the 90-day ALDS with the mRS was very strong, with *r* = −0.95. The concurrent validity of the ALDS with the 90-day NIHSS and BI was similarly very strong: NIHSS-ALDS, *r* = −0.87; BI-ALDS, *r* = 0.93.

**Figure 1:**
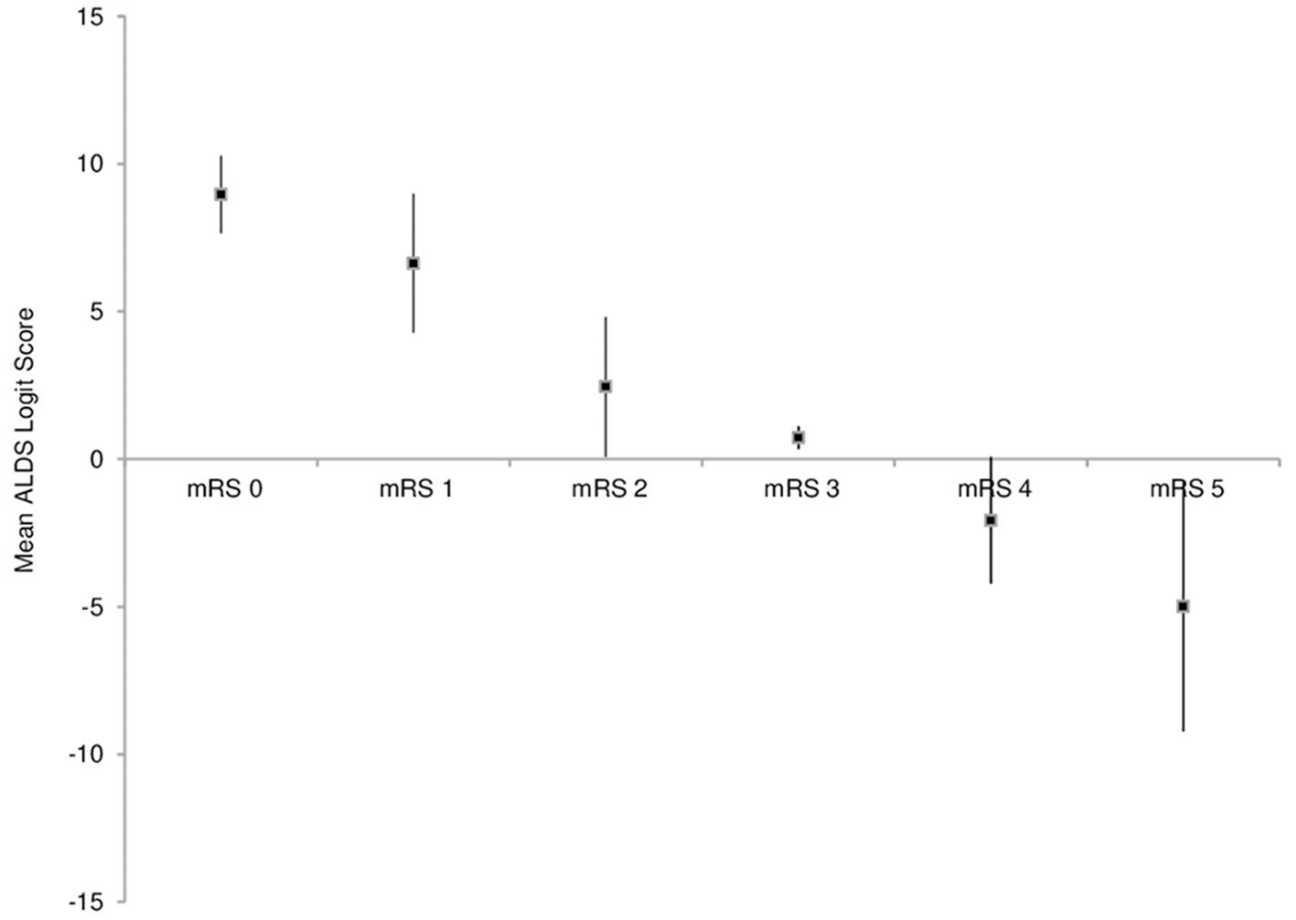
Dot plot of mean (±95%CI) ALDS logit values for each of mRS grades 0–5.

Table 3 shows the mean ALDS logit and transformed scores for each of the seven Rankin levels. There was a linear relation between the mRS ordinal ranks and mean ALDS logit scores (Figure 1), confirmed by a spline vs. linear fit showing that the relationship between mRS vs. ALDS logit is not significantly different from linear (p = 0.95). The average change in ALDS logit for a one-unit change in mRS was 2.8 ± 0.21 (least square regression slope ± SE).

**Table 3:** ALDS scores associated with each modified Rankin Scale level at 90 days.

**Modified Rankin Scale**	**ALDS logit score; mean (SD)**	**ALDS transformed score; mean (SD)**
0 No symptom (*n =* 12)	8.96 (2.06)	99.32 (2.37)
1 No sign of disability (*n =* 12)	6.63 (3.70)	95.52 (7.25)
2 Slight disability (*n =* 3)	2.45 (0.95)	90.20 (7.18)
3 Moderate disability (*n =* 9)	0.72 (0.51)	66.44 (11.20)
4 Moderate severe disability (*n =* 6)	−2.08 (2.04)	21.43 (29.21)
5 Severe disability (*n =* 6)	−4.99 (4.03)	2.82 (1.39)
6 Death (*n =* 7)	−7.70 (–)	0 (–)

Scale granularity was superior for the ALDS as compared with the mRS. The average number of patients with a particular scale score value was less for the ALDS than the mRS, 1.9 (SD ± 3.2) vs. 8.0 (SD ± 3.6), *p* < 0.001.

**Figure 2:**
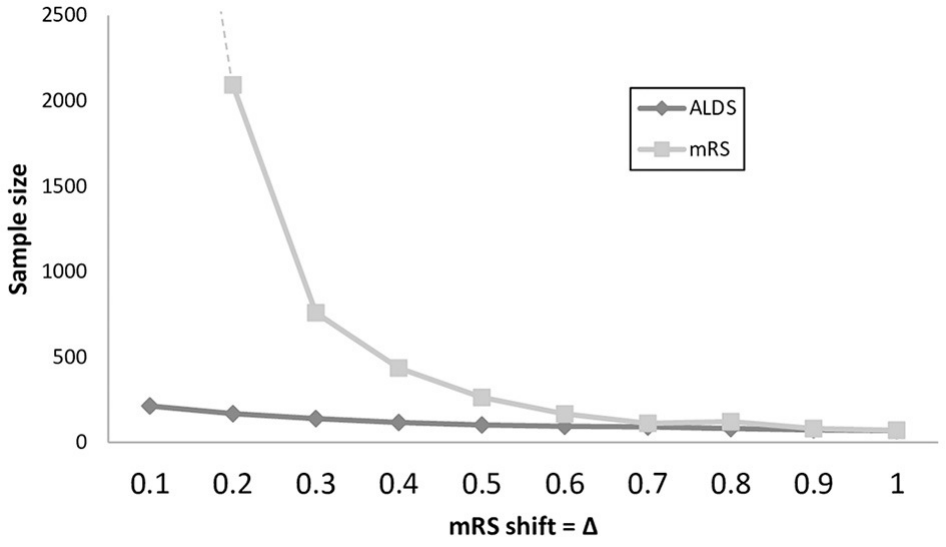
Line graph showing sample sizes to detect treatment effects in trials over a range of treatment effect magnitudes, with power 0.80 and alpha 0.05. Extent of shift on the mRS scale of the treatment is shown on the X axis and is varied in 0.1 increments from modest (0.1 shift) to substantial (1.0 shift). For both small and moderate treatment effects, trials that use the ALDS rather than the mRS as the primary trial endpoint have substantially smaller sample size requirements.

For the trial sample size analysis, since the relationship between mRs and ALDS logit is not significantly different from being linear, the power calculations were computed assuming that ALDS logit increases by 2.8 units on average for each 1.0 unit decrease in mRS. As shown in Figure 2 and Supplementary Table S1, over a wide range of mild-to-moderate treatment magnitudes, trials using the ALDS rather than the mRS as the primary endpoint afford greater power to detect actual present therapy benefits. When a treatment exerted a benefit by shifting the mRS by 0.2 to 0.6, required sample sizes were 2 to 12-fold smaller using the ALDS rather than the mRS.

## Discussion

This study found that, among patients enrolled in an acute stroke treatment trial, the Academic Linear Disability Score exhibited excellent convergent validity with the current standard, the modified Rankin Scale and demonstrated advantages in greater granularity and scale linearity. The ALDS additionally demonstrated good internal validity in the stroke population and excellent concurrent validity with measures of other aspects of stroke functional change. The greater precision and responsiveness of the ALDS increased the projected power of clinical trials to detect treatment effects, reducing needed sample sizes.

This study extends the important work of Weisscher et al. in characterizing the performance of ALDS in stroke populations (13). While their study analyzed an all-comer stroke patient population, this study evaluated patients enrolled in an actual stroke trial. Since trial patients typically are more homogenous than general practice populations, our results have particular relevance for trial design uses of the ALDS. Similarly, this study assessed outcomes 3 months post-stroke, the most common timepoint for primary outcome assessment in acute stroke trials (1), while the prior study assessed outcomes 6 months after stroke. In addition, the current study directly assessed the trial power impact of the use of the ALDS vs. the mRS, which was not a topic of the prior investigation.

The internal validity performance of the ALDS was less strong in the current study than in the study of Weisscher et al., with the observed order of item difficulty for our stroke population differing a bit more from assigned item difficulty scores. Several factors may have contributed to this difference, including sociocultural activity differences between American (current study) and European (prior study) patients and families and potential framing effects of other assessments (mRS, BI, and NIHSS) performed in our study immediately prior to performing the ALDS.

This study may usefully be contrasted with another recent initiative to place global disability scores within a continuous numeric framework—the utility-weighted modified Rankin Scale (UW-mRS) (10). The UW-mRS is designed to mitigate interpretative difficulties related to the ordinal nature of the standard modified Rankin Scale by assigning the health-related quality of life weights to each mRS level. The weighting is derived from two approaches to valuing the mRS scale: (1) assigning utility values by mapping the mRS to EuroQol scale tariffs and (2) assigning disability weights by using the person tradeoff method of the World Health Organization Global Burden of Disease project (19, 20).

On the UW-mRS, the original mRS levels have a non-interval distribution, with the seven mRS levels grouped into four clusters, 0–1, 2–3, 4, and 5–6. In contrast, along the linear disability dimension defined by the ALDS, mRS ranks 0–5 are fairly evenly arrayed. Whereas the utility and disability weight methods map the mRS to how much a health state is valued by patients and providers, the ALDS method maps the mRS to a dimension defined by increasing physical and cognitive task difficulties. These findings are consonant with the conceptual understanding that the ALDS maps a disability spectrum while the UW-mRS maps a disability-related quality of life spectrum.

A theoretical concern is that a highly granular scale such as the ALDS may detect treatment effects that are statistically significant but not clinically significant (21). This is not an issue for the mRS, for which at least 5 of the 6 single-step scale changes are highly meaningful to patients, including 0 to 1 (no vs. some symptoms), 1 to 2 (able vs. not able to work), 2 to 3 (able vs. not able to live alone), 3 to 4 (ambulatory vs. non-ambulatory), and 4 to 5/6 (not requiring constant nursing care vs. requiring constant care or dead). While the minimally clinical important difference (MCID) for the ALDS has not been well-defined, the MCID for the mRS has been characterized. When comparing the average mRS of treatment group 1 vs. treatment group 2, the MCID has been shown to be at most 0.05 and as low as 0.013 (22, 23). Accordingly, all of the range of effect magnitudes we explored, assessing interventions that would benefit the experimental population by shifting its average mRS score by all 0.1 increments from 0.1 to 1.0, exceeded the MCID for the mRS. The ALDS outperformed the mRS in requiring smaller sample sizes over a wide range of mild-to-moderate effect magnitudes that are highly relevant to stroke clinical practice. The span in which the ALDS was more efficient includes the great preponderance of treatment benefit magnitudes that have been detected in actual acute stroke clinical trials and generally accepted in the clinical community as reflecting clinically meaningful change, including intravenous thrombolysis in the 0–3 h window (mRS shift 0.53) (24), intravenous thrombolysis in the 3–4.5 h window (mRS shift 0.21) (25), and intravenous thrombolysis beyond 4.5 h with imaging selection (mRS shift 0.15) (26). However, the ALDS did not offer greater efficiency in detecting very large treatment benefit magnitudes such as those for endovascular thrombectomy (mRS shift 0.73) (27).

While the ALDS and the mRS are both designated as disability assessments, the aspects of functioning they assess are not entirely coterminous. The ALDS assesses basic, instrumental, and extended activities of daily living. The mRS assesses these aspects but also the presence of symptoms and societal roles. The ALDS accordingly measures disability as it was conceived in the World Health Organization definition of 1980–2001, which distinguished between disability, impairment, and handicap (28). The mRS measures disability as conceived in the World Health Organization definition in use since 2001 which includes all three of these features as aspects of disability (29).

Our study has limitations. The original sample size was modest, limiting study generalizability. The patients were enrolled in a hyperacute, ambulance-based stroke trial and may differ from patients enrolled in more broad-based trials. Our cohort was recruited from multiple hospitals but in a single geographic region. Although our patient population exhibited substantial race-ethnic diversity and stroke subtype diversity, further studies in additional clinical trial populations are needed. It is possible that the correlation between the mRS and the ALDS was overestimated because the ALDS question sets given to each patient were based on the mRS score; however, each question set included some queries that were well above and below the typical response range to minimize any such bias. It is a general principle of administration of item response bank tests that selection occurs based on the most appropriate items for each person so that assessment precision is optimized for a given test length and irrelevant items can be avoided (30). The approach taken here was pragmatic, allowing paper administration of the ALDS rather than computerized adaptive testing that can be challenging to implement in large stroke clinical trials (15–17). A similar approach was actually the initial method of ALDS administration when the scale was first developed (31, 32). This general administration strategy has recently been re-endorsed as a practical means of item bank testing deployment (18). Our sample size analyses are based on a modest patient sample and on simulated treatment effects; analyses are needed of larger populations of patients enrolled in an actual trial of treatment with beneficial effects and administered both the ALDS and mRS.

We used only one among the several approaches in the literature for performing modified Rankin Scale scoring. In addition to the Rankin Focused Assessment, available methods include (1) unstructured, holistic rank assignment (33), (2) non-operationalized scoring after videotape training (34), (3) the simplified modified Rankin Scale Questionnaire (35), (4) the mRS structured interview (36), and (5) the mRS-9Q (37). Compared with other approaches, the Rankin Focused Assessment has shown generally better inter-rater reliability, is an objective clinician-reported rather than subjective patient-reported assessment, or both. The advantages of the ALDS may be greater when compared with other, less reliable methods of mRS ascertainment.

We conclude that the Academic Medical Center Linear Disability Score is well-correlated with the modified Rankin Scale in patients enrolled in an acute stroke trial. The use of disability scales such as the ALDS, which are more fine-grained and interval rather than ordinal in character, can potentially increase the power of acute stroke clinical trials.

## Data availability statement

Two datasets were used in this study. The publicly available dataset of the NIH FAST-MAG can be found here: https://www.ninds.nih.gov/current-research/research-funded-ninds/clinical-research/archived-clinical-research-datasets. The additional dataset of ALDS scores is available to investigators upon review and approval of the FAST-MAG Publications Committee. Requests to access the additional dataset of ALDS scores should be directed to the corresponding author JS.

## Ethics statement

The studies involving human participants were reviewed and approved by UCLA IRB. The patients/participants provided their written informed consent to participate in this study.

## Author contributions

NC performed data analysis and draft writing. SS, FC, and JS performed data collection. RC performed supervision. JG and SH performed statistical analysis. JS performed draft writing. All authors reviewed and provided draft comments. All authors contributed to the article and approved the submitted version.
