# Supplementary material for: Staged use of ordinal and linear disability scales: a practical approach to granular assessment of acute stroke outcome
Source: Front Neurol. 2023 Jun 28;14:1174686. doi: 10.3389/fneur.2023.1174686 (PMC10344771; doi:10.3389/fneur.2023.1174686)
Supplement: Supplementary file 1 [file Data_Sheet_1.pdf]

Online Supplemental Table S1. Sample sizes for 80% power to detect treatment groups differences.

delta R= true mRS shift	ALDS sample size	mRS sample size	mRS/ALDS ratio
0.10	213	>3000	>14.08
0.20	168	2093	12.46
0.30	139	759	5.46
0.40	117	436	3.73
0.50	103	263	2.55
0.60	94	167	1.78
0.70	91	112	1.23
0.80	82	121	1.48
0.90	74	81	1.09
1.00	69	71	1.03
