# Supplementary material for: Staged use of ordinal and linear disability scales: a practical approach to granular assessment of acute stroke outcome
Source: Front Neurol. 2023 Jun 28;14:1174686. doi: 10.3389/fneur.2023.1174686 (PMC10344771; doi:10.3389/fneur.2023.1174686)
Supplement: Supplementary file 2 [file Data_Sheet_2.pdf]

Supplementary Methods Text

The following 4 pages contain:

1) Administration instructions
2) The 5 ALDS question sets, each containing 15 items indexed to a particular mRS score level

AMC Linear Disability Score (ALDS) after mRS Assessment

Introduction

Assessing the clinically meaningful effect on outcome in stroke patients allows more accurate interpretation in clinical trials. The AMC (Academic Medical Center) Linear Disability Score (ALDS) is a calibrated generic item bank to measure the level of physical disability in patients.(1-3) The ALDS is a sensitive and generic disability scale which measures a broader range of activities compare to the Rankin assessment. The major difference between the ALDS and the Rankin assessment is that the ALDS is a linear disability score assessment and is a patient-reported outcome whereas the Rankin score is an ordinal disability score assessment and is a rater-assigned outcome.

General Instructions

Sources of Information
Obtain information from the same people that provided answers for the Rankin Focused Assessment (RFA).

Choose the Right Assessment
This assessment is intended for use after completing the Rankin Focused Assessment (RFA). There are different sets of questions matched to each Rankin score result. Choose the AMC Linear Disability Score (ALDS) question set according to the Rankin score. For example, if your patient has Rankin score of 3, then assess ALDS 3 question set. However, if every question in the initial ALDS question set ends up marked 'Yes' or 'NA', then please proceed to next lower numbered ALDS question set and complete the assessment *unique* to that ALDS set. For example, if your patient has a Rankin score of 2, then assess ALDS 2. However, if the patient answered all the questions in ALDS 2 'Yes' or 'NA' then proceed to ALDS 1 and assess all the *unique* questions in ALDS 1 that were not in ALDS 2. Similarly, if every question in the initial ALDS question set ends up marked 'No' or 'NA', then please proceed to next higher numbered ALDS question set and complete the assessment *unique* to that ALDS set. For example, if your patient has a Rankin score of 2, then you will complete ALDS 2. If the patient answered all questions in ALDS 2 'No' or 'NA' then proceed to ALDS 3 and complete all the *unique* questions in ALDS 3.

Rankin Score	Assessment
Rankin Score 0	ALDS 1
Rankin Score 1	ALDS 1
Rankin Score 2	ALDS 2
Rankin Score 3	ALDS 3
Rankin Score 4	ALDS 4
Rankin Score 5	ALDS 5

Scoring Instructions
There are two response options which are 'Yes' and 'No'. If a patient responds that he/she can carry out the activity but only with difficulty, then 'Yes' should be marked. If a patient says the question is not applicable because the patient has never attempted the activity in the question after stroke, then mark the 'NA' box. The answer should reflect what the patient can do without assistance of another person. However, assistance of a walker, cane and other prosthetic devices are fine.

References

1. *Holman R, Lindeboom R, Glas C, Vermeulen M, de Haan RJ. Constructing an item bank using item response theory: the AMC linear disability score project. Health Serv Outcomes Res Methodol. (2003) 4:19–33.*
2. *Holman R, Lindeboom R, Vermeulen M, de Haan RJ. The AMC Linear Disability Score project in a population requiring residential care: psychometric properties. Health Qual Life Outcomes. (2004) 2:42. doi: 10.1186/1477-7525-2-42*
3. *Weisscher N, Vermeulen M, Roos YB, de Haan RJ. What should be defined as good outcome in stroke trials; a modified Rankin score of 0-1 or 0-2? J Neurol. (2008) 255:867–74.*

Visit Nurse Name : ____

Patient Number : ______________ Subject Initials : __ __ __ Date of Visit : __ / __ / __

AMC Linear Disability Score Rating Form

Information for completing this form was obtained from (please check all that apply) :

□ Patient	□ Sister
□ Spouse	□ Brother
□ Son	□ Other relative, specify relationship : ________________
□ Daughter	□ Friend
□ Father	□ Nurse
□ Mother	□ Home health aide
□ Physical therapist	□ Occupational therapist
□ Speech therapist	□ Physician

□ Other individual, specify role: ________________

Please mark (X) in the appropriate box. Choose the questionnaire according to the Rankin Score. Please assess all the questions. See the instruction sheets for further information.

**ALDS 1 : Assessment for Patient with Rankin Score 0 or Rankin Score 1**				
		Yes	No	NA
1	Can you vacuum a flight of stairs?	□	□	□
2	Can you carry a bag of shopping upstairs?	□	□	□
3	Can you go for a walk in the woods?	□	□	□
4	Can you travel by local bus?	□	□	□
5	Can you carry a tray?	□	□	□
6	Can you walk up a hill?	□	□	□
7	Can you cut your toe nails?	□	□	□
8	Can you stand for 10 minutes?	□	□	□
9	Can you use a washing machine?	□	□	□
10	Can you walk down a flight of stairs?	□	□	□
11	Can you go for a short walk (15 min)?	□	□	□
12	Can you change the sheets on a bed?	□	□	□
13	Can you buy a few things from the store?	□	□	□
14	Can you take a shower and wash your hair?	□	□	□
15	Can you pick something up from the floor?	□	□	□

Visit Nurse Name : ____

Patient Number : ______________ Subject Initials : __ __ __ Date of Visit : __ / __ / __

Please mark (X) in the appropriate box. Choose the questionnaire according to the Rankin Score. Please assess all the questions. See the instruction sheets for further information.

**ALDS 2 : Assessment for Patient with Rankin Score 2**				
		Yes	No	NA
1	Can you vacuum a flight of stairs?	□	□	□
2	Can you carry a bag of shopping upstairs?	□	□	□
3	Can you go for a walk in the woods?	□	□	□
4	Can you travel by local bus?	□	□	□
5	Can you carry a tray?	□	□	□
6	Can you go shopping for clothes?	□	□	□
7	Can you cut your toe nails?	□	□	□
8	Can you stand for 10 minutes?	□	□	□
9	Can you use a washing machine?	□	□	□
10	Can you walk down a flight of stairs?	□	□	□
11	Can you change the sheets on a bed?	□	□	□
12	Can you buy a few things from the store?	□	□	□
13	Can you take a shower and wash your hair?	□	□	□
14	Can you pick something up from the floor?	□	□	□
15	Can you prepare breakfast or lunch?	□	□	□

**ALDS 3 : Assessment for Patient with Rankin Score 3**				
		Yes	No	NA
1	Can you travel by local bus?	□	□	□
2	Can you carry a tray?	□	□	□
3	Can you cut your toe nails?	□	□	□
4	Can you stand for 10 minutes?	□	□	□
5	Can you walk down a flight of stairs?	□	□	□
6	Can you go for a short walk (15 min)?	□	□	□
7	Can you change the sheets on a bed?	□	□	□
8	Can you take a shower and wash your hair?	□	□	□
9	Can you pick something up from the floor?	□	□	□
10	Can you get in and out of a car?	□	□	□
11	Can you prepare breakfast or lunch?	□	□	□
12	Can you put on/take off socks and slippers?	□	□	□
13	Can you sit on the edge of a bed from lying down?	□	□	□
14	Can you put on and take off a coat?	□	□	□
15	Can you walk to and get on and off the toilet?	□	□	□

Visit Nurse Name : ____

Patient Number : ______________ Subject Initials : __ __ __ Date of Visit : __ / __ / __

Please mark (X) in the appropriate box. Choose the questionnaire according to the Rankin Score. Please assess all the questions. See the instruction sheets for further information.

**ALDS 4 : Assessment for Patient with Rankin Score 4**				
		Yes	No	NA
1	Can you carry a tray?	□	□	□
2	Can you walk up a flight of stairs?	□	□	□
3	Can you buy a few things from the store?	□	□	□
4	Can you take a shower and wash your hair?	□	□	□
5	Can you pick something up from the floor?	□	□	□
6	Can you get in and out of a car?	□	□	□
7	Can you peel and core an apple?	□	□	□
8	Can you prepare breakfast or lunch?	□	□	□
9	Can you eat a meal at the table?	□	□	□
10	Can you put on/take off socks and slippers?	□	□	□
11	Can you put pants on?	□	□	□
12	Can you sit on the edge of a bed from lying down?	□	□	□
13	Can you put on and take off a coat?	□	□	□
14	Can you walk to and get on and off the toilet?	□	□	□
15	Can you wash your lower body when taken to sink?	□	□	□

**ALDS 5 : Assessment for Patient with Rankin Score 5**				
		Yes	No	NA
1	Can you change the sheets on a bed?	□	□	□
2	Can you pick something up from the floor?	□	□	□
3	Can you get in and out of a car?	□	□	□
4	Can you peel and core an apple?	□	□	□
5	Can you eat a meal at the table?	□	□	□
6	Can you put on/take off socks and slippers?	□	□	□
7	Can you sit up (from lying) in bed?	□	□	□
8	Can you get a book off the shelf?	□	□	□
9	Can you answer the telephone?	□	□	□
10	Can you make a bowl of cereal?	□	□	□
11	Can you move between two dining chairs?	□	□	□
12	Can you wash and dry your lower body?	□	□	□
13	Can you put on and take off a coat?	□	□	□
14	Can you wash and dry your face and hands?	□	□	□
15	Can you get out of bed into a chair?	□	□	□
